# Converting Cells into Cellular Factories

**DOI:** 10.5936/csbj.201210001

**Published:** 2012-11-08

**Authors:** Hal S. Alper

**Affiliations:** a Department of Chemical Engineering, The University of Texas at Austin, 200 E Dean Keeton St. Stop C0400, Austin, TX 78712; b Institute for Cellular and Molecular Biology, The University of Texas at Austin, 2500 Speedway Avenue, Austin, TX 78712

This special issue focusses on the theme of metabolic engineering and the design of cellular factories. This is a very timely issue given that we sit at a newly developing nexus between systems biology understanding and synthetic biology design and construction [1]. The tangible result of these forces is a newfound capacity to rapidly produce an expanding array of new molecules in recombinant organisms [2]. It was not long ago that the list of metabolic engineering feats was small and limited to mostly natural metabolites such as ethanol, lysine, glutamic acid, and carotenoids. Now, this list includes molecules that can be used as fuels, polymer precursors, pharmaceuticals, and commodity/specialty chemicals. Both the number and chemical diversity of molecules continues to increase rapidly. We are quickly moving to an era where it is conceivable to produce any desired organic molecule in a cellular system. The papers in this special issue highlight the rapid expansion of both product and organismal diversity that is being seen in research across the field. The early years of metabolic engineering were focused on rewiring endogenous metabolism to overproduce native metabolites such as ethanol and amino acids. Now, we must tackle a wider array of non-native metabolites including large-chain alcohols, complex polymers, and natural products. These feats require sophisticated genetic control along with new enzymes and pathways (both imported as well as synthetic). At the organismal level, our systems biology understanding and advanced molecular biology has enabled our move beyond model organisms and into exotic yeasts, photosynthetic organisms, and even into electrotrophs. While these organisms certainly raise new challenges for modern molecular biology, they provide promising biotechnological traits. The thought of forming a new company around a novel organism with the hopes of being able to eventually perform the required genetic engineering used to be very risky. Now, such a proposition is quite commonplace.

As stated above, we are entering a time when systems biology is truly beginning to collide with synthetic biology. Put another way, our understanding of cellular complexity is beginning to be matched with our ability to engineer synthetic metabolism and regulation. This combination of strengths will be quite fruitful for the field of metabolic engineering. For example, several articles in this issue take advantage of genome scale models for the prediction of metabolic engineering targets. Systems level understanding of cellular function and automated methods for developing genome scale models rapidly expands the metabolic engineering toolkit. The progression of identifying a unique strain, sequencing the genome, and assembling a genome-scale model has progressed rapidly in the past few years and has become financially and intellectually tangible, even for a single researcher. Until recently, such intricate knowledge of cellular metabolism has been limited to only well-studied model organisms. Integrating this information in the form of a genome-scale model helps researchers parse the interconnectedness of cellular metabolism. These models both predict genetic modification as well as establish bounds of metabolism. The latter can help in selecting the ideal organism for a given task.

Beyond model-guided metabolic engineering, we are beginning to witness an expanded capacity to engineer synthetic function in cells. The interface of synthetic biology with metabolic engineering offers the ability to design control system, create new metabolic pathways, and import metabolic modules. This work is also inherent in some of the papers in this special issue. In particular, the rapid expansion in our capacity to make natural products and non-native molecules is predicated by our ability to import and control synthetic metabolic pathways. These skills have enabled the rapid expansion we see in the molecules produced by cellular factories.

The themes of systems biology and synthetic biology are interwoven throughout the papers for this special issue. They represent the state-of-the-art in designing, optimizing, and engineering cells. The end goal of transforming cellular systems into a cellular factory is not always easy to attain. Yet, an increase in our capacity to understand and alter cells continues to make this feat ever-attainable. The rapid expansion in both molecules produced and cell types engineered shows the promise inherent in the goals of metabolic engineering. In time, these approaches will lead to a new industrial biotechnological revolution in which all organic molecules of interest can be made via cellular processes. Given the great strides taking place in the field, that time is not too far away.
